# Computational epigenetic landscape analysis reveals association of CACNA1G-AS1, F11-AS1, NNT-AS1, and MSC-AS1 lncRNAs in prostate cancer progression through aberrant methylation

**DOI:** 10.1038/s41598-022-13381-0

**Published:** 2022-06-17

**Authors:** Mahafujul Islam Quadery Tonmoy, Atqiya Fariha, Ithmam Hami, Kumkum Kar, Hasan Al Reza, Newaz Mohammed Bahadur, Md Shahadat Hossain

**Affiliations:** 1 Department of Biotechnology & Genetic Engineering, Noakhali Science and Technology University, Noakhali, Bangladesh; 2 Department of Genetic Engineering and Biotechnology, University of Dhaka, Dhaka, Bangladesh; 3 Department of Applied Chemistry and Chemical Engineering, Noakhali Science and Technology University, Noakhali, Bangladesh; 4 Computational Biology and Chemistry Lab (CBC), Noakhali Science and Technology University, Noakhali, Bangladesh

**Keywords:** Cancer, Computational biology and bioinformatics

## Abstract

Aberrant expression of long non-coding RNAs (lncRNAs), caused by alterations in DNA methylation, is a driving factor in several cancers. Interplay between lncRNAs’ aberrant methylation and expression in prostate cancer (PC) progression still remains largely elusive. Therefore, this study characterized the genome-wide epigenetic landscape and expression profiles of lncRNAs and their clinical impact by integrating multi-omics data implementing bioinformatics approaches. We identified 62 differentially methylated CpG-sites (DMCs) and 199 differentially expressed lncRNAs (DElncRNAs), where 32 DElncRNAs contain 32 corresponding DMCs within promoter regions. Significant negative correlation was observed between 8 DElncRNAs-DMCs pairs. 3 (cg23614229, cg23957912, and cg11052780) DMCs and 4 (CACNA1G-AS1, F11-AS1, NNT-AS1, and MSC-AS1) DElncRNAs were identified as high-risk factors for poor prognosis of PC patients. Overexpression of hypo-methylated CACNA1G-AS1, F11-AS1, and NNT-AS1 and down-regulation of hyper-methylated MSC-AS1 significantly lower the survival of PC patients and could be a potential prognostic and therapeutic biomarker. These DElncRNAs were found to be associated with several molecular functions whose deregulation can lead to cancer. Involvement of these epigenetically deregulated DElncRNAs in cancer-related biological processes was also noticed. These findings provide new insights into the understanding of lncRNA regulation by aberrant DNA methylation which will help to clarify the epigenetic mechanisms underlying PC.

## Introduction

Prostate cancer (PC) is the second most commonly diagnosed cancer in males globally accounting for 1,276,106 new cases and 358,989 deaths (3.8% of all cancer-related deaths in men) in 2018^1^. However 2,293,818 additional cases are expected till 2040 and there will also be a minor difference in mortality (an increase of 1.05%)^2^. This indicates that despite the majority of PC patients experiencing a slow tumor progression, a certain percentage of the cases discovered are more aggressive and lethal cancer variants^3^.

The earlier studies on PC were condensed on suggesting a significant heterogeneity in structural alterations of the genome-such as the variations in DNA copy numbers, the electron transport system (ETS) of transcription factors causing genetic fusions within the tumors and in gene expression profiles which were found in around half of the prostate tumors^4–12^. The diversity in genomic expressions and variations in the tumor behavior in cases of PC were attributed to some other factors involving genomic aberrations^13^. Epigenetic alterations have been more immediately charged with being responsible as an early event leading up to further somatic and genetic mutation in several tumors^14^. The event of DNA methylation is one of the most popularly studied epigenomic changes^15^ that have been found to regulate gene expression, thereby impacting tumorigenesis and cancer progression^16–22^ in both metastatic and locally advanced tumors^23,24^. About 60% of human gene promoters have been reported to be overlapped with CpG islands (CGIs)^25^—the small clusters of residues where DNA methylation occurs^26^. Such affiliation of the CGI with the DNA promoters leads to methylation of the promoter regions of the DNA which ultimately ends up in gene silencing^27^. The simple mechanism for the process can be summed up as methylation of the 5′ carbon of cytosine in CGIs of gene promoters. Any alteration in DNA methylation processes can lead to cancer initiation, progression, invasion and metastasis^28,29^.

Noncoding RNA, earlier regarded as ‘transcriptional noise’ of the genome, has successively acquired the recognition for its important functional involvement in a variety of biological processes, including gene expression regulation, alternative splicing regulation, cellular structure formation, and so on^30,31^. Long non-coding RNAs containing more than 200 nucleotides in length are also members of non-coding RNAs which have recently emerged as a class of tumor-suppressor and oncogenic genes^32,33^. The effects of lncRNAs on carcinogenesis may be performed through the process of transcriptional, post-transcriptional and epigenetic modification^33^. Aberrant expression of lncRNA contributes to the development and progression of cancers which demonstrate the potential role of lncRNAs as novel diagnostic and prognostic biomarkers for cancer and therapeutic targets as well^34,35^. MiRNAs, another member of non-coding RNAs, have been extensively investigated to identify miRNA/miRNA-targeted signatures to improve the diagnosis and prognosis of several cancers^36–40^. Emerging evidences have documented the superiority of lncRNA as diagnostic and prognostic biomarkers for cancers compared to miRNAs^29,41,42^. However, the detailed function of lncRNAs in development and progression of cancer is still unknown^43^. Therefore, concurrent studies have also shown how DNA methylation of lncRNA-encoding genes can affect the downstream targets^29^.

The hyper-methylation of the CGIs in some of the tumor suppressor genes has been frequently noticed in cases of PC tumors as well^44,45^. Previous studies on DNA methylation in PC fundamentally used the CpG islands array on either the thousands of genomic regions around the methylation site or the promoter regions^46,47^. However, interrelation between the DNA methylation and the lncRNA expression in PC still remain largely unknown. In this present study, a genome-wide integrated analysis between the DNA methylation and the expression of lncRNA was performed to characterize the correlation between the DNA methylation and lncRNA regulation, thereby figure out the epigenetically regulated lncRNAs. Moreover, potential clinical relevance of these epigenetically regulated lncRNAs with the PC patients’ survival was also investigated. Further to that, molecular functions of these lncRNAs along with the biological processes in which they are involved were also elucidated.

## Results

### Characterization of DNA methylation pattern in prostate cancer

**Figure 1:**
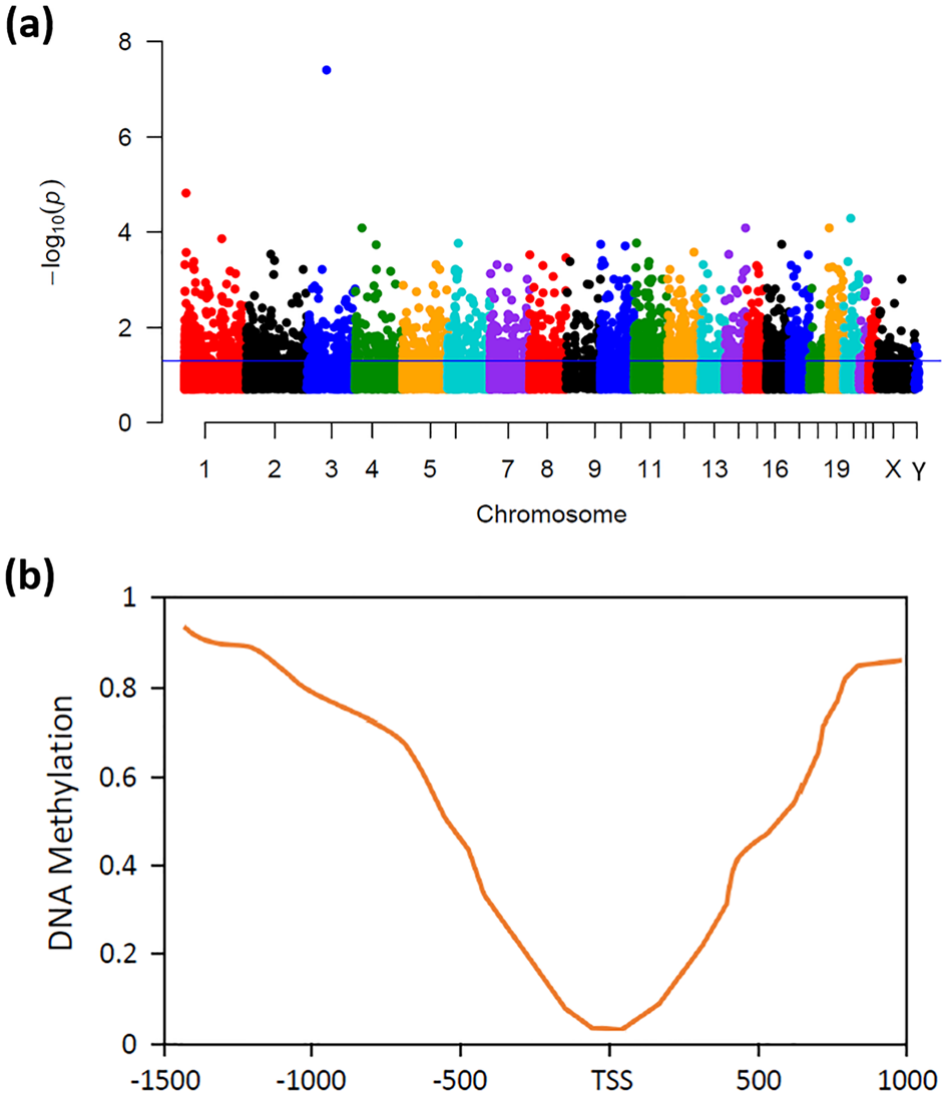
DNA methylation pattern of the CpG sites in prostate cancer. (**a**) Distribution of the CpG sites across the chromosomes. The horizontal straight line (blue) represents the 10^−1.3^ (0.05) threshold on the p value. CpG sites that cross the threshold value are considered significant. (**b**) Methylation distribution around the lncRNAs ranging from 1.5 kb upstream to 1 kb downstream of the transcription start site (TSS). Y-axis represents the beta values of the identified CpG sites in Prostate cancer.

We conducted a differential methylation analysis and long non-coding RNAs **(**lncRNA) annotation to identify the differentially methylated CpG sites (DMCs) in prostate cancer patients which are located in the promoter regions of lncRNAs. Regarding this, initially, we obtained 23 methylation array data series from the GEO database among a total of 2030 data series for Prostate cancer. Among the 23 methylation array data series, an independent methylation cohort GSE112047 (contains 31 tumor and 16 control samples) satisfied all the inclusion criteria specified in the method and selected for differential methylation analysis. We then computed differential methylation patterns between 31 tumor and 16 control samples and identified a total of 18,066 CpG sites where 2622 were identified with FDR < 0.05 (Fig. 1a). From this figure, it can be noticed that the CpG sites are distributed in all the chromosomes. 566 of the 2622 CpG sites were found to localize in the lncRNA promoter regions and designated for assessing the DNA methylation alterations within the lncRNA promoter regions (Table S1). Methylation distribution (beta value) of the 566 CpG sites in lncRNA promoter regions manifested a relatively higher methylation density at the distal regions of the lncRNA promoter regions than at Transcription Start Site (TSS) (Fig. 1b). Among the 566 CpG sites, 62 were identified to have an absolute delta-beta value > 0.2 and considered as DMCs. Besides, with a 0.2 cut off size, 6 differentially methylated regions (DMRs) were identified among a total of 340 methylated regions. TCGA (PRAD)-450k methylation array was used to validate the DNA methylation patterns of the 566 CpG sites and found consistency with our results.

### Characterization of differentially expressed lncRNAs in prostate cancer

**Figure 2:**
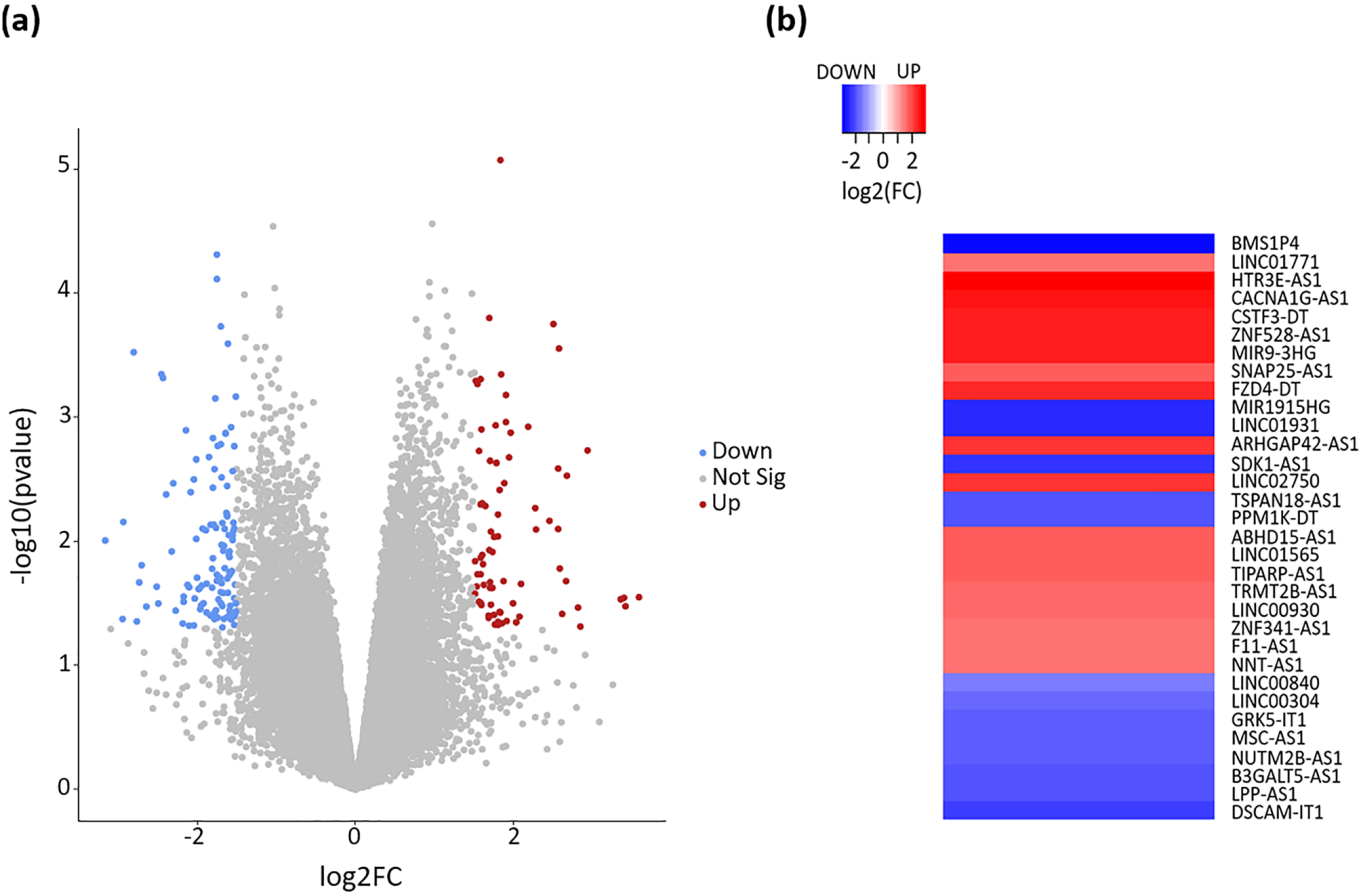
Differential expression signature of lncRNAs in prostate cancer. (**a**) Volcano plot shows the expression pattern of the lncRNAs in Prostate cancer. The red and blue dots indicate the significantly (p-value < 0.05) up-regulated (log2FC > 1.5) and down-regulated (log2FC < − 1.5) lncRNAs, respectively. (**b**) Heatmap shows the differentially expressed lncRNAs whose promoter region gets methylated differentially in Prostate cancer. The red and blue bars respectively represent the significantly (p-value < 0.05) up-regulated (log2FC > 1.5) and down-regulated (log2FC < − 1.5) lncRNAs.

To identify the differentially expressed lncRNAs, an independent lncRNA cohort GSE140927 (contains 4 tumor and 4 control samples) was retrieved from the GEO database amongst a total of 114 non-coding RNA data series. This analysis identified 26,246 lncRNAs after removing the duplicates and the Circular RNAs (circRNAs) from the list. 199 (78 up-regulated and 121 down-regulated) of the 26,246 lncRNAs were identified with P-value < 0.05 and |log2FC| > 1.5 and considered as significant and differentially expressed lncRNA (DElncRNA) (Fig. 2a). Further to that, 32 (18 up-regulated and 14 down-regulated) (Fig. 2b) out of 199 DElncRNAs were sorted as they contain corresponding 32 (out of 62) DMCs (identified in the previous step) within their promoter regions (Table S2). Moreover, these 32 DElncRNAs were categorized based on their position in the genome and found that these DElncRNAs were at four categories of different genomic locations, i.e. antisense RNA, Long intergenic non-coding RNA, divergent transcript, and intronic transcript. Antisense RNA, long intergenic non-coding RNA, divergent transcript, and intronic transcript were accounted for 17, 10, 3, and 2, respectively.

### Correlation analysis between the DElncRNAs and the corresponding DMCs

**Figure 3:**
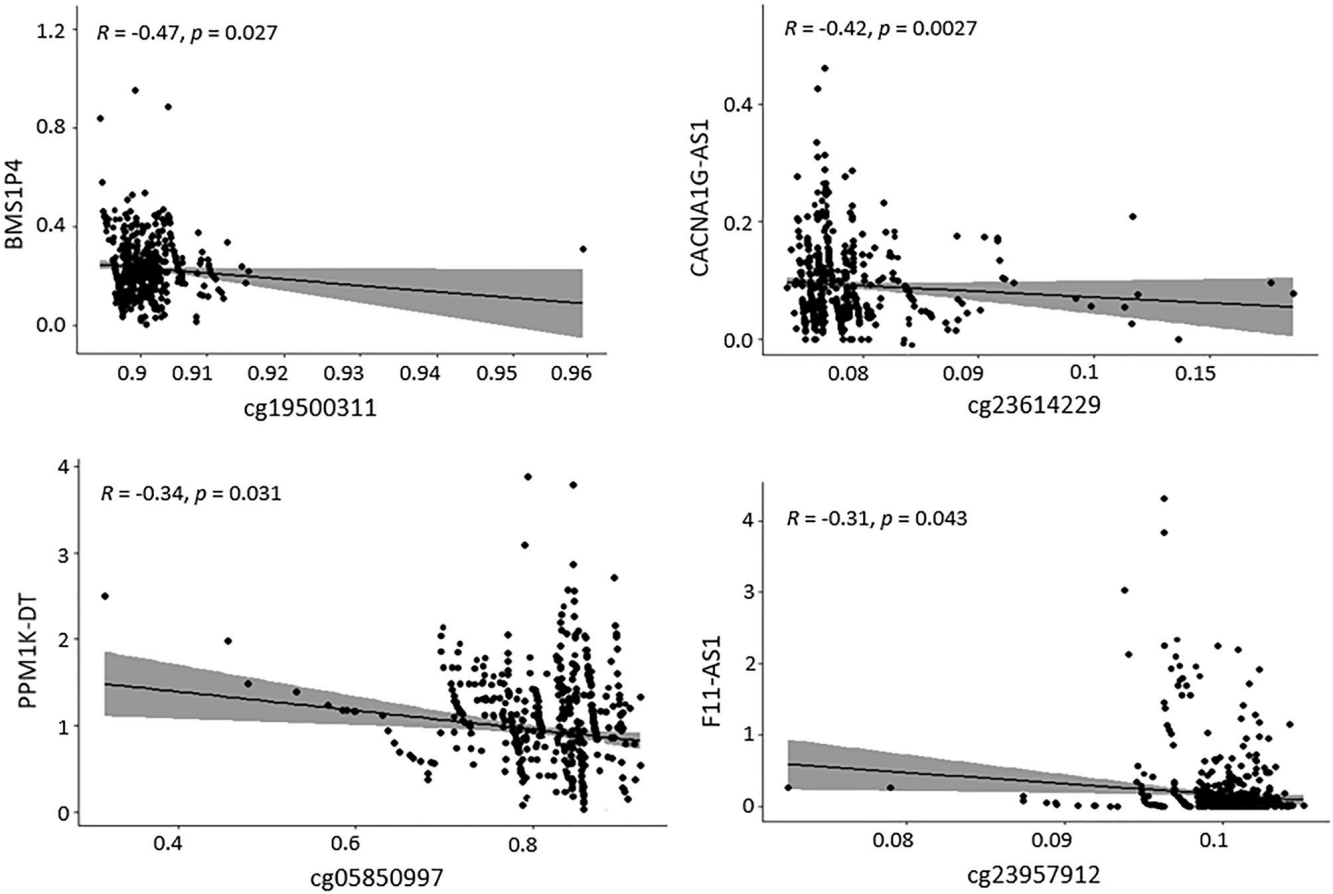
Correlation between the DMCs (cg19500311, cg23614229, cg05850997, and cg23957912) and the expression of lncRNAs (BMS1P4, CACNA1G-AS1, PPM1K-DT, and F11-AS1) in matched samples. The beta values of the DMCs are depicted in X-axis and the expression (FPKM) values of lncRNAs are depicted in Y-axis. *R* and *p* indicate the Spearman's correlation coefficient and the p-values derived from Spearman's correlation, respectively.

**Figure 4:**
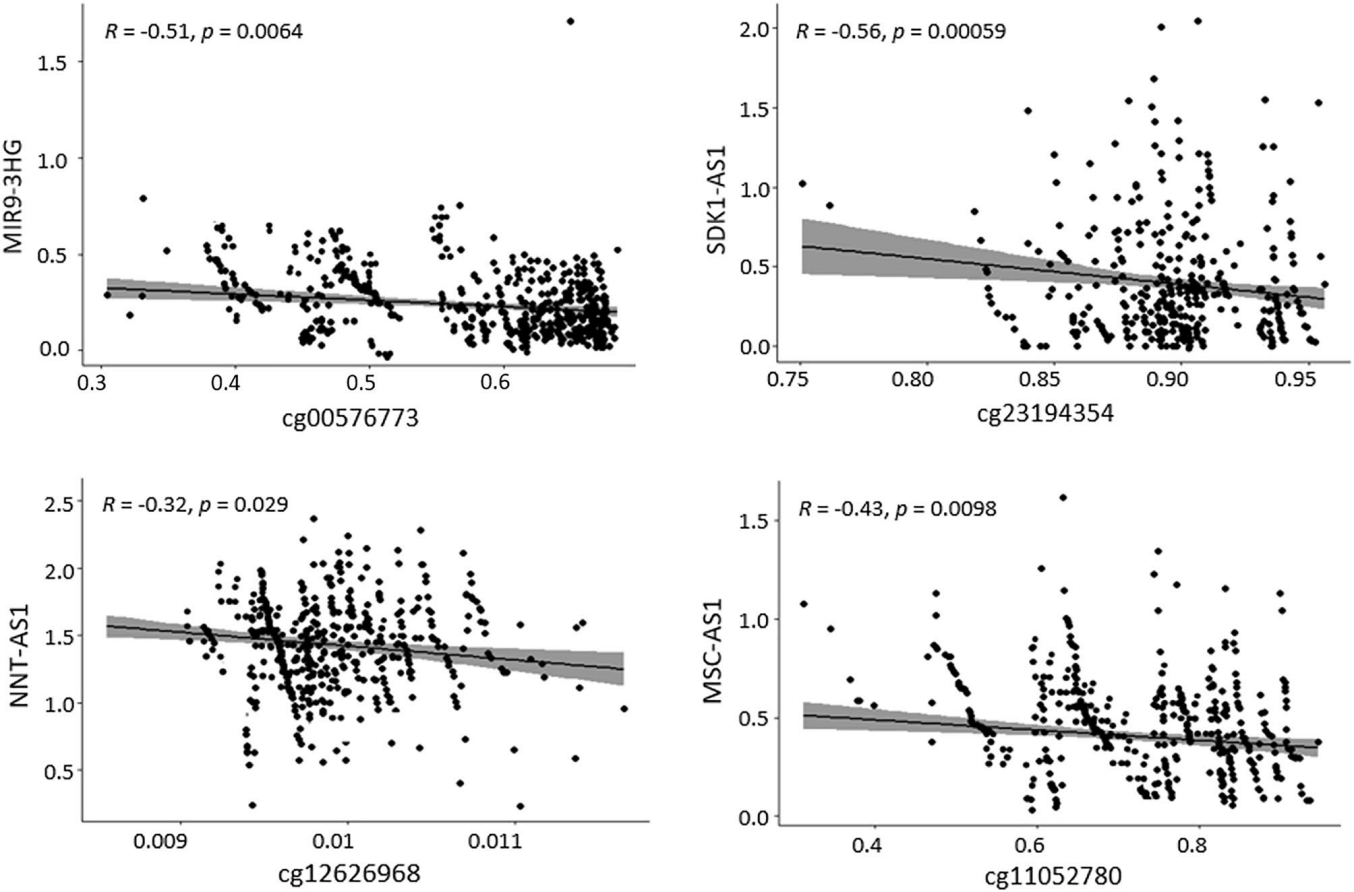
Correlation between the DMCs (cg00576773, cg23194354, cg12626968, and cg11052780) and the expression of lncRNAs (MIR9-3HG, SDK1-AS1, NNT-AS1, and MSC-AS1) in matched samples. The beta values of the DMCs are depicted in X-axis and the expression (FPKM) values of lncRNAs are depicted in Y-axis. *R* and *p* indicate the Spearman's correlation coefficient and the p-values derived from Spearman's correlation, respectively.

We combined the expression pattern of the DElncRNAs and the methylation pattern of the DMCs to determine whether the expression of the 32 DElncRNAs is modulated by the methylation of the corresponding 32 CpG sites. Concerning this, we performed Spearman's correlation analysis between these 32 pairs (DElncRNAs-DMCs) omics data and found 24 negatively and 8 positively correlated pairs (Table S3). Out of 24 pairs, a significant negative correlation [correlation coefficient (R) > − 0.3 and p-value < 0.05] was observed between the 8 DElncRNA (BMS1P4, CACNA1G-AS1, MIR9-3HG, SDK1-AS1, PPM1K-DT, F11-AS1, NNT-AS1, and MSC-AS1) and their respective 8 DMCs (cg19500311, cg23614229, cg00576773, cg23194354, cg05850997, cg23957912, cg12626968, and cg11052780) (Figs. 3 and 4). The down-regulation of BMS1P4, SDK1-AS1, PPM1K-DT, and MSC-AS1 was significantly associated with the hyper-methylation of their respective CpG sites cg19500311, cg23194354, cg05850997, and cg11052780 which were found within the promoter region of these DElncRNAs. Conversely, the up-regulation of CACNA1G-AS1, MIR9-3HG, F11-AS1, and NNT-AS1 is significantly associated with the hypo-methylation of their respective CpG sites cg23614229, cg00576773, cg23957912, and cg12626968 which were found within the promoter region of these DElncRNAs.

### A consequence of aberrant DNA methylation and expression of DElncRNA on the survival of prostate cancer patients

**Figure 5:**
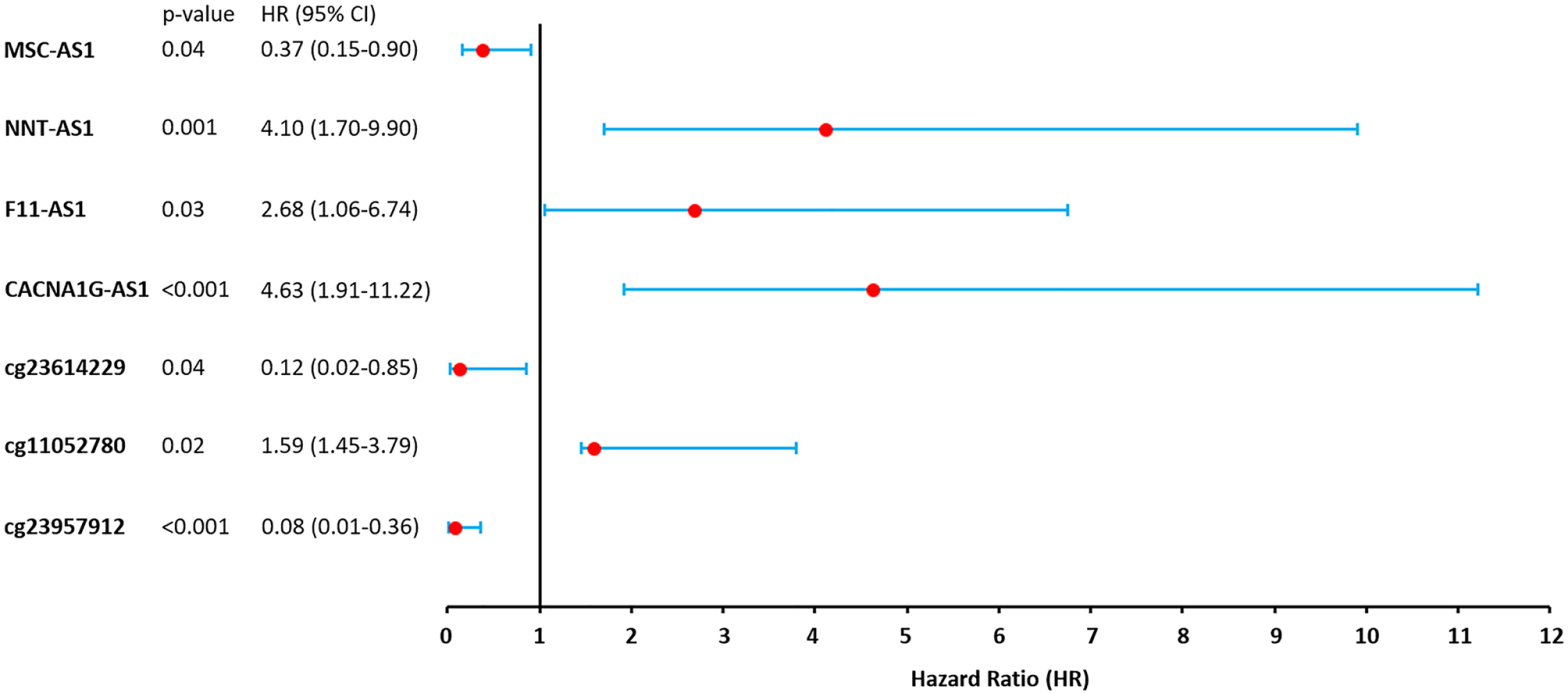
Forest plot showing the correlation between the methylation of DMCs and expression of DElncRNAs with the overall survival of the Prostate cancer patients. Median beta value for the DMCs and the median FPKM value for the lncRNAs were considered as cut-off value.

**Figure 6:**
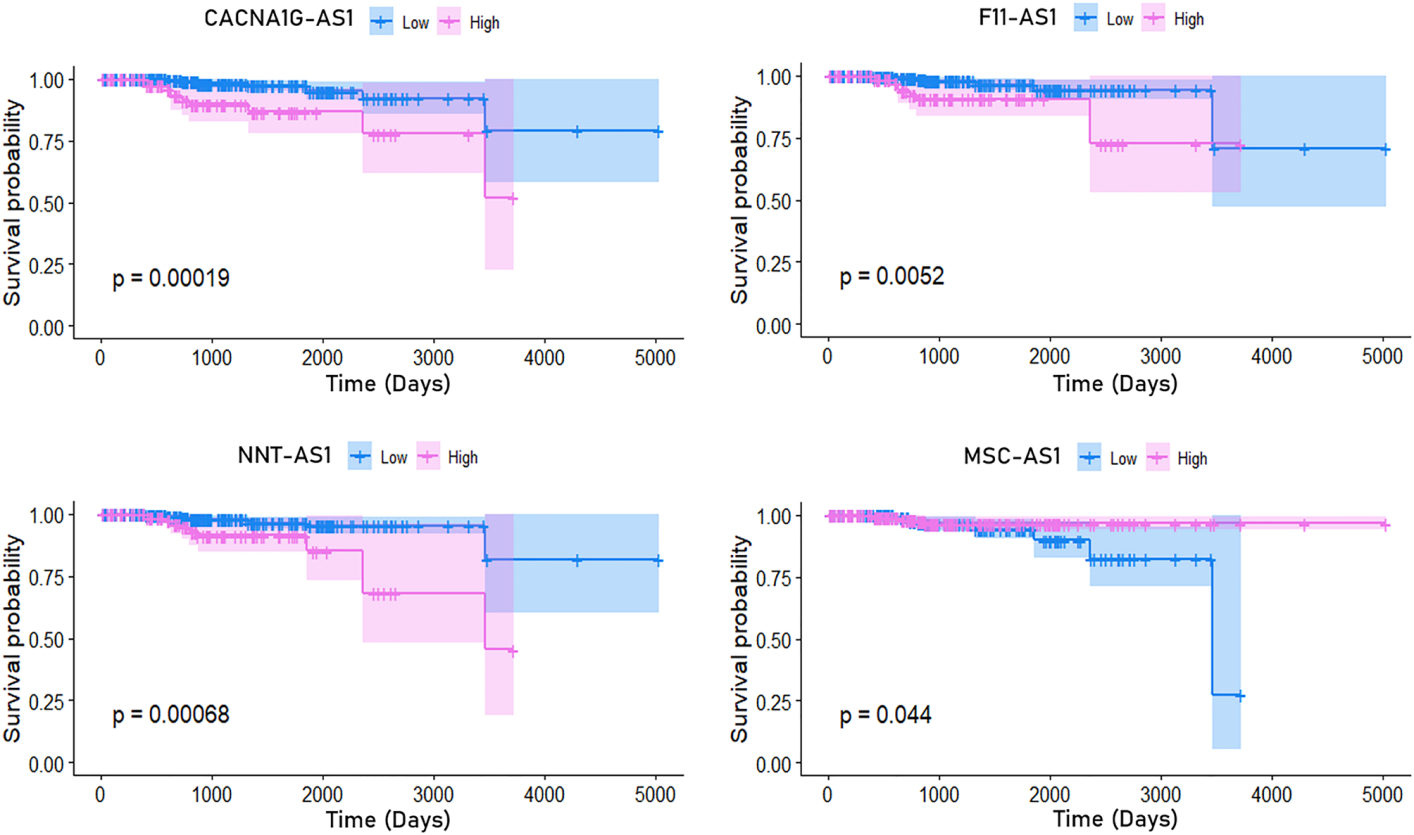
Overall survival analysis of the patients with prostate cancer based on the expression of CACNA1G-AS1, F11-AS1, NNT-AS1, and MSC-AS1. Median FPKM value was considered as cut-off value.

The methylation pattern of the 8 DMCs and the expression of the 8 DElncRNAs in prostate cancer patients were evaluated to determine their impact on the patient’s survival. Univariate Cox regression analysis identified 3 (cg23614229, cg23957912, and cg11052780) DMCs and 4 (CACNA1G-AS1, F11-AS1, NNT-AS1, and MSC-AS1) DElncRNAs as high-risk factors (95% CI HR ⊉ 1 and p-value < 0.05) for the prognosis of patients with Prostate cancer (Fig. 5). Hypo-methylation of two DMCs cg23614229 (within the promoter region of CACNA1G-AS1), and cg23957912 (within the promoter region of F11-AS1) were found to significantly responsible for the poor prognosis of the patients with Prostate cancer (95% CI HR < 1 and p-value < 0.05). Conversely, hyper-methylation of the cg11052780 (within the promoter region of MSC-AS1) probe was found to have significant effect on poor prognosis of the patients with Prostate cancer (95% CI HR > 1 and p-value < 0.05) (Fig. 5). Higher expression (95% CI HR > 1) of CACNA1G-AS1, F11-AS1, and NNT-AS1 were found to significantly lower the overall survival rates of the patients with Prostate cancer than those of patients with lower expression of these DElncRNAs (Figs. 5 and 6). On the contrary, poor overall survival was remarked for the patients with low expression (95% CI HR < 1) of MSC-AS1 compared with patients with high expression of MSC-AS1 (Figs. 5 and 6). To avoid the dependency only on the expression of DElncRNAs, additionally, multivariate Cox regression analysis was also done by adjusting other covariates (patients age and primary Gleason grade) with the DElncRNAs’ expression and the results still showed that the expression of these 4 DElncRNAs was significantly correlated with poor survival (95% CI HR ⊉ 1 and p-value < 0.05) of the patients with Prostate cancer (Table 1).

**Table 1:** Cox regression analysis (univariate and multivariate) of the variables associated with the overall survival of the prostate cancer patients.

Variables	Univariate Cox regression analysis			Multivariate Cox regression analysis		
	Hazard ratio (HR)	95% confidence interval (CI)	P-value	Hazard ratio (HR)	95% confidence interval (CI)	P-value
Age (> 61/ < 61)	1.15	0.83–1.62	0.01	1.59	1.11–2.26	0.01
Primary Gleason grade (Pattern 3 + Pattern 4 + Pattern 5)	1.23	0.69–2.25	0.02	1.79	1.14–2.80	0.04
CACNA1G-AS1 (high expression/low expression)	4.63	1.91–11.22	< 0.001	6.02	1.70–21.86	0.04
F11-AS1 (high expression/low expression)	2.68	1.06–6.74	0.03	5.09	3.08–8.33	0.03
NNT-AS1 (high expression/low expression)	4.10	1.70–9.90	0.001	4.33	2.62–7.98	0.001
MSC-AS1 (high expression/low expression)	0.37	0.15–0.90	0.04	0.32	0.12–0.83	0.01

### Functional enrichment analysis

**Figure 7:**
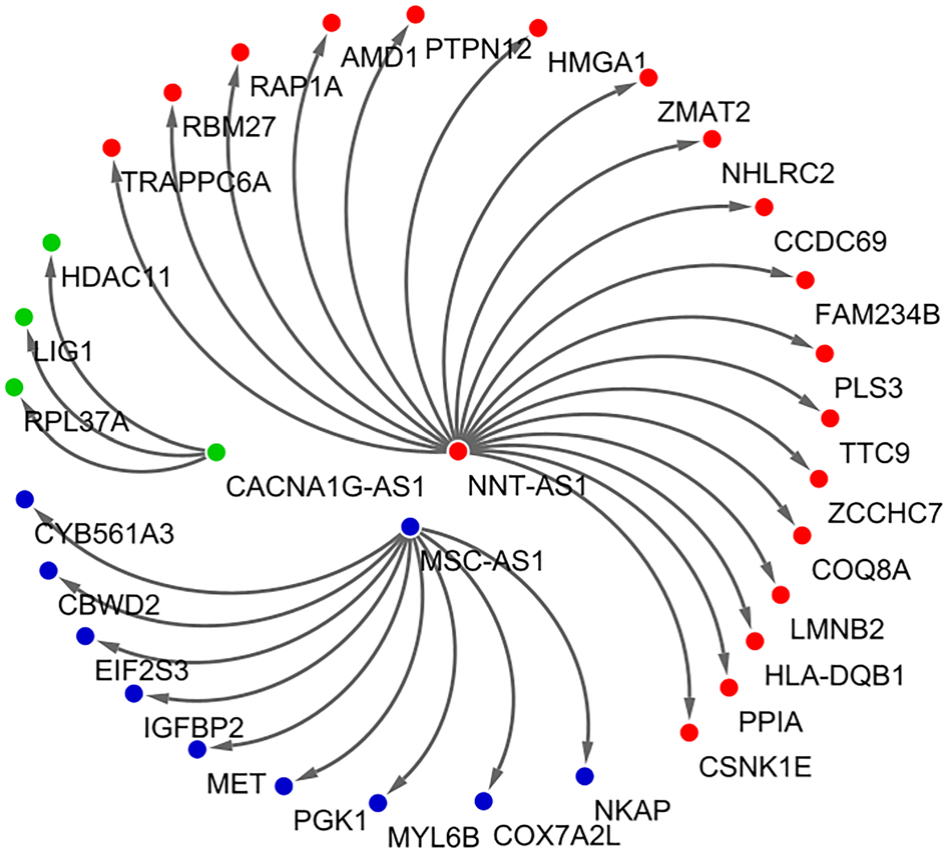
lncRNA-mRNA interaction network showing protein-coding genes targeted by the CACNA1G-AS1, NNT-AS1, and MSC-AS1.

**Figure 8:**
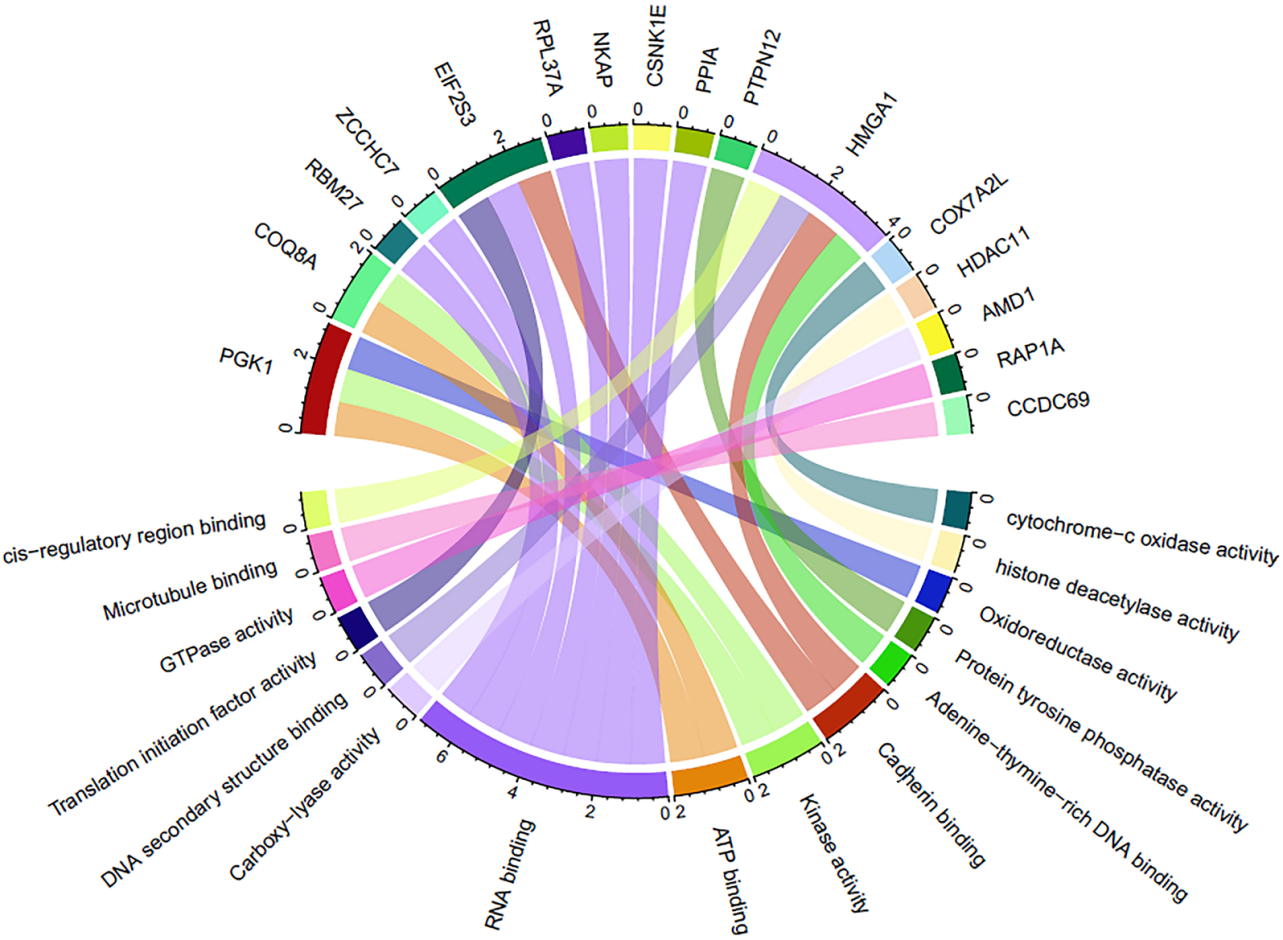
Gene ontology (GO)-molecular function (MF) analysis of the CACNA1G-AS1, NNT-AS1, and MSC-AS1 targeted protein-coding genes.

**Figure 9:**
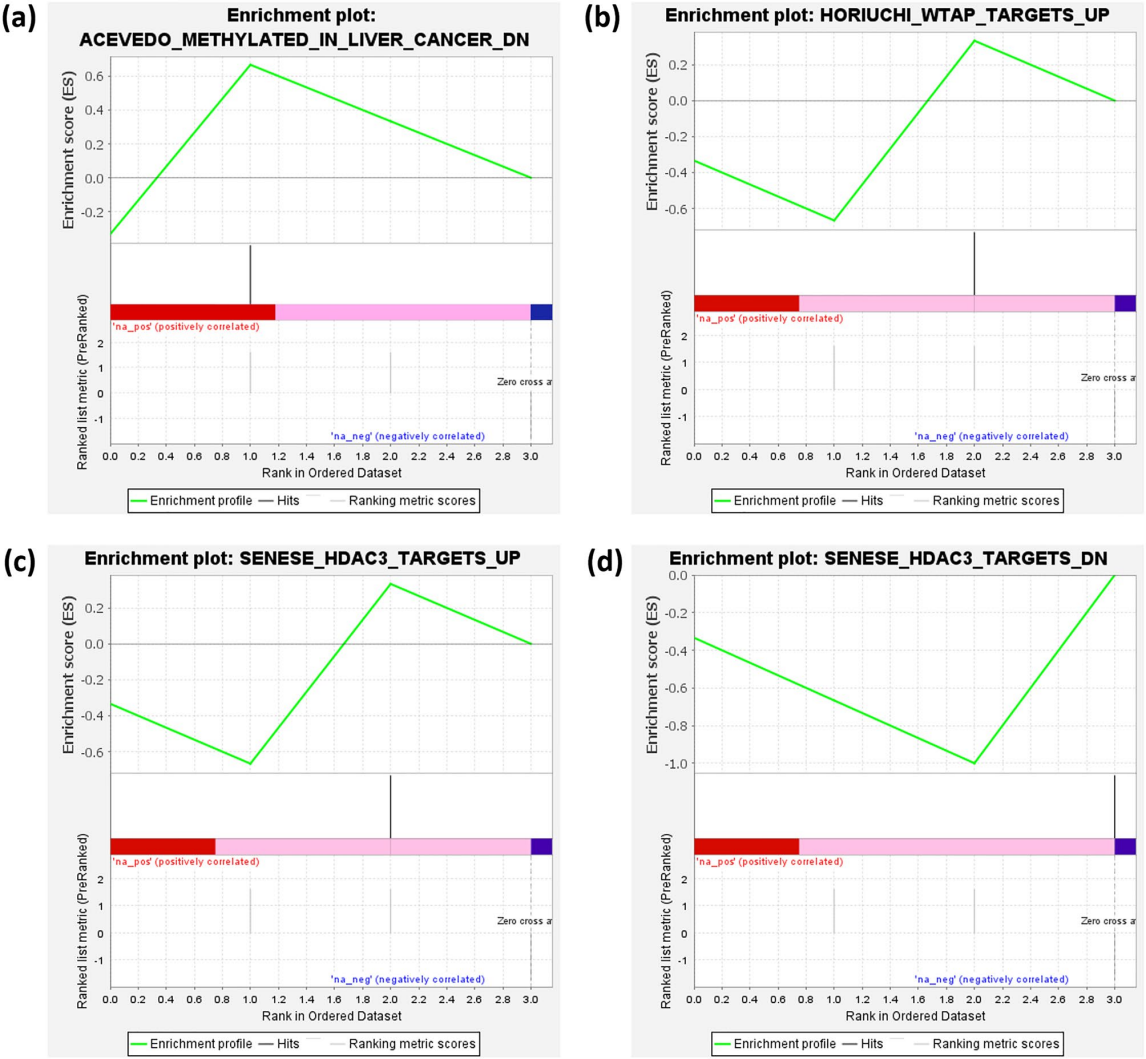
Gene set enrichment analysis (GSEA) exhibiting the biological process associated with the (**a**) F11-AS1, (**b**) CACNA1G-AS1, (**c**) NNT-AS1, and (**d**) MSC-AS1. A positive enrichment score represents positive correlation with the phenotype profile where a negative score represents inverse correlation with the phenotype profile. Red, pink and blue color indicate the high, moderately high, and low expression.

Functional enrichment analysis was accomplished to identify the potential molecular functions and the underlying biological processes in which CACNA1G-AS1, F11-AS1, NNT-AS1, and MSC-AS1 are involved. In terms of this, lncRNA-mRNA interaction network was determined to figure out the protein-coding genes whose expression can be modulated by the CACNA1G-AS1, F11-AS1, NNT-AS1, and MSC-AS1 lncRNAs. This analysis identified a total of 30 protein-coding genes, 18 of which can be modulated by the NNT-AS1, whereas MSC-AS1, and CACNA1G-AS1 can modulate 9, and 3 protein-coding genes, respectively (Fig. 7). We did not find any protein-coding gene associated with the F11-AS1. These 30 protein-coding genes were assigned for characterizing their molecular functions and only 16 protein-coding genes were found to have significant (P-value < 0.05) association with several molecular functions (Fig. 8). NNT-AS1 targeted RBM27, RAP1A, AMD1, PTPN12, HMGA1, CCDC69, ZCCHC7, COQ8A, PPIA and CSNK1E genes were pointed out to involve in RNA binding, GTPase activity, carboxy-lyase activity, protein tyrosine phosphatase activity, cis-regulatory region binding, DNA secondary structure binding, cadherin binding, adenine–thymine rich DNA binding, Microtubule binding, kinase activity, and ATP binding (Fig. 8). RNA binding, cytochrome-c-oxidase activity, ATP binding, kinase activity, oxidoreductase activity, translation initiation factor activity, and cadherine binding molecular functions were found to actualize by the MSC-AS1 targeted NKAP, COX7A2L, PGK1, EIF2S3 genes (Fig. 8). CACNA1G-AS1 targeted RPL37A, HDAC11 genes were identified to associate with RNA binding, and histone deacetylase activity (Fig. 8). Additionally, gene set enrichment analysis (GSEA) was executed to identify the biological processes in which these lncRNAs are involved. A functionally unknown lncRNA, F11-AS1, was explored and found that F11-AS1 positively correlated with the ACEVEDO_METHYLATED_IN_LIVER_CANCER_DN set, means up-regulation of the F11-AS1 positively correlates with the hypo-methylation of the genes associated in liver cancer (Fig. 9a). CACNA1G-AS1 negatively correlated with the HORIUCHI_WTAP_TARGETS_UP set, means down-regulation of the CACNA1G-AS1 negatively correlates with the up-regulation of the Wilms' tumor 1-associating protein (WTAP) targeted genes (Fig. 9b). The “SENESE_HDAC3_TARGETS_DN” set is enriched in the NNT-AS1 low expression group which suggests that the downregulation of this lncRNA negatively correlates with the downregulation of Histone Deacetylase 3 (HDAC3) targeted genes (Fig. 9c). MSC-AS1 negatively correlated with the SENESE_HDAC3_TARGETS_UP set which indicates that the downregulation of this lncRNA negatively correlates with the up-regulation of HDAC3 targeted genes (Fig. 9d).

## Discussion

A relatively new ncRNA class, lncRNAs, have been characterized as an important kind of gene expression regulator and can play crucial roles in carcinogenesis^48^. In recent years, lncRNAs have become a research hotspot for study in a variety of cancer fields. It has already been demonstrated that the lncRNAs are shedding new insight into understanding the cancer pathways and their potential role as novel diagnostic and predictive cancer biomarkers in clinical practice^49,50^. Aberrant expression of lncRNAs has also been found to be associated with prostate cancer (PC) emergence and progression, and several lncRNAs have been identified as diagnostic and predictive biomarker for PC^51,52^. Emerging evidence indicates that methylation of DNA is a key epigenetic regulator of the expression of lncRNAs, and epigenetic alterations might interfere with the lncRNAs expression profile which can promote cancer^53–55^. However, interrelation of the aberrant methylation in the promoters of lncRNAs with the emergence and progression of PC still remains largely elusive. Therefore, in this present study, we conducted an integrated analysis of DNA methylation and the expression of lncRNAs to characterize the dysregulated lncRNAs in the development and progression of PC. Furthermore, we explored the interplay between the biological and clinical relationships of the lncRNAs with the prognosis of PC patients.

A genome-wide methylation alteration was observed in lncRNA promoter regions which revealed a relative reduction of the methylation density at the TSS during the development and progression of PC. Mapping of the aberrant DNA methylation to the promoter regions of lncRNAs identified a total of 32 epigenetically deregulated DElncRNAs in PC, where 18 and 14 were found to be up-regulated and down-regulated, respectively. A significant inverse correlation was found between the promoter methylation (cg19500311, cg23614229, cg00576773, cg23194354, cg05850997, cg23957912, cg12626968, and cg11052780) of 8 corresponding aberrantly expressed DElncRNAs (BMS1P4, CACNA1G-AS1, MIR9-3HG, SDK1-AS1, PPM1K-DT, F11-AS1, NNT-AS1, and MSC-AS1). DNA methylation in promoters is inversely correlated with the corresponding gene expression^56^. The hyper-methylation of CpG sites cg19500311, cg23194354, cg05850997, and cg11052780 significantly lower the expression of the corresponding BMS1P4, SDK1-AS1, PPM1K-DT, and MSC-AS1 lncRNAs. Conversely, significant up-regulation of the CACNA1G-AS1, MIR9-3HG, F11-AS1, and NNT-AS1 lncRNAs is caused by the hypo-methylation of the respective CpG sites cg23614229, cg00576773, cg23957912, and cg12626968. These DElncRNAs were divided into two groups based on their methylation patterns and expression levels to distinguish PC patients with different prognoses and thereby demonstrate a potential function of the anomalously methylated DElncRNAs in the survival of PC patients. From an integrated analysis of multi-omics and clinical data, prognosis prediction is a key factor for understanding the biological complexity of PC. The cg23614229, cg23957912, and cg11052780 DMCs and the CACNA1G-AS1, F11-AS1, NNT-AS1, and MSC-AS1 DElncRNAs were identified as significant high-risk factors for the poor prognosis of PC patients. Higher expression of the hypo-methylated CACNA1G-AS1, F11-AS1, and NNT-AS1 significantly lower the overall survival rates of the PC patients. CACNA1G-AS1 was demonstrated to have significantly higher expression in several cancer, such as ovarian cancer and non-small cell lung cancer^57,58^. Pre-ranked GSEA analysis showed that the high expression of CACNA1G-AS1 might be associated with the up-regulation of the Wilms’ tumor 1-associating protein (WTAP) targeted genes. Overexpression of WTAP contributes to aggressive features of numerous cancers such as renal cell carcinoma, acute myeloid leukemia, diffuse large B-cell lymphoma, cholangiocarcinoma, hepatocellular carcinoma and play a role as oncogene^59–63^. Previous studies also showed the higher expression of NNT-AS1 in numerous cancers such as cholangiocarcinoma, osteosarcoma, non-small cell lung cancer, colorectal cancer, including prostate cancer^64–68^. The increased expression of NNT‑AS1 positively correlates with the up-regulation of Histone Deacetylase 3 (HDAC3) targeted genes as predicted by the GSEA analysis. Previous findings showed that the overexpression of HDAC3 acts as an oncogenic feature and can promote the progression of cholangiocarcinoma and gastric cancer^69,70^. Unlike CACNA1G-AS1 and NNT‑AS1, the up-regulation of F11-AS1 has been found to be associated with the suppression of liver hepatocellular carcinoma^71,72^. GSEA analysis revealed that up-regulation of the F11-AS1 was likely to be associated with the hypo-methylation of the genes associated in liver cancer. As up-regulation of F11-AS1 significantly lower the overall survival of PC patients, its overexpression feature could be a potential diagnostic and prognostic biomarker for PC. Low expression of hyper-methylated MSC-AS1 significantly lower the overall survival of the patients with PC. As GSEA results predicted, the down-regulation of MSC-AS1 might be involved in the up-regulation of Histone Deacetylase 3 (HDAC3) targeted genes. Function enrichment analysis of mRNAs regulated by these four epigenetically deregulated lncRNAs uncovers a new perception of the potential functional relevance of these DElncRNAs. We found that these DElncRNAs are involved in many molecular functions such as RNA binding, GTPase activity, protein tyrosine phosphatase activity, DNA secondary structure binding, cadherin binding, kinase activity, cytochrome-c-oxidase activity, translation initiation factor activity, and histone deacetylase activity. Deregulation of such molecular functions can result in several disease conditions including but not limited to cancer^73–77^. Hence, aberrant expression of these epigenetically deregulated lncRNAs might cause the distortion of these cellular functions which can lead to the emergence and progression of PC.

The results provide by the current study is an evidence of the genome-wide alteration of DNA methylation of lncRNAs in PC patients. The candidate epigenetically deregulated lncRNAs CACNA1G-AS1, F11-AS1, NNT-AS1, and MSC-AS1 might function as key regulatory factors in the development and progression of PC and could be potential therapeutic and prognostic biomarkers for PC, which were associated with the poor prognosis of the patients with PC. The present results will help to unravel a more detailed understanding of the aberrant methylation patterns of lncRNAs and thereby clarify the epigenetic mechanisms underlying PC.

## Conclusion

The current study investigated the crosstalk between the DNA methylation and expression of lncRNAs and their impact on the clinical prognosis of PC patients by integrating multi-omics data using several bioinformatics approaches. The establishment of a detailed understanding of DNA methylation-altered CACNA1G-AS1, F11-AS1, NNT-AS1, and MSC-AS1 lncRNAs in PC will facilitate the characterization of oncogenic lncRNAs. The mechanistic and functional characterization of these epigenetically deregulated lncRNAs may help to reveal the path of future development of lncRNA-based PC specific therapies. The significant prognostic association of CACNA1G-AS1, F11-AS1, NNT-AS1, and MSC-AS1 lncRNAs suggests their association in PC progression which will shed light on the future development of lncRNA-based prognostic biomarkers specific for PC. Since the results provided by this present study are based on in silico analysis, further in-depth experimental investigations are required to validate the findings.

## Methods

### Characterization of DNA methylation pattern in prostate cancer

A differential methylation analysis and long non-coding RNAs **(**lncRNA) annotation was performed to identify the prostate cancer (PC) related differentially methylated CpG sites (DMCs) and differentially methylated regions (DMRs) located in the promoter regions of lncRNAs. The GEO (Gene Expression Omnibus) database^78^ was explored to search for methylation array datasets for prostate cancer. This database is the most widely used public repository for accessing raw, processed, and descriptive gene expression data, as well as other functional genomics data sets including genome methylation, genome variation and genome-protein interaction^78^. The keyword “prostate cancer” was used for this search and then considered the following criteria for choosing the appropriate datasets (i) the methylation array dataset were restricted to “*Homo sapiens*”, (ii) the experiment type was specified to “Methylation profiling by array”, (iii) dataset having tumor and control samples, (iv) “Illumina HumanMethylation450 BeadChip” was nominated as methylation profiling platform, and (v) the dataset must contain “IDAT” files. Afterwards, the R package “minfi”^79^ was used to process the Illumina methylation 450K array data to identify the DMCs and DMRs between PC samples and adjacent tissues. “dmpFinder” function (type = categorical) of this package was utilized to identify the DMCs where significance level was set as absolute delta beta value > 0.2 (20% difference on beta value) and P-value < 0.05. The Benjamini and Hochberg technique was used to determine the false discovery rate (FDR) from multiple testing adjustments of raw P-value. “bumphunter” function (resamples = 100, cut off = 0.2) of the “minfi” package was then used to identify the DMRs. The “HM450.hg38.manifest” file (https://zwdzwd.github.io/InfiniumAnnotation) was used to perform the genomic annotation of each CpG site. According to the “HM450.hg38.manifest” and GENCODE v36 (https://www.gencodegenes.org/human/release_36.html) reference annotation file, the genomic coordinates of each lncRNA was obtained. We then combined both the information regarding the genomic coordinates of CpG sites and lncRNAs to look for the differentially methylated loci inside the promoter regions [2500 bp upstream and 1000 bp downstream from the putative transcription start site (TSS)] of lncRNAs. “qqman”^80^ R package was used to construct a Manhattan plot to represent the chromosomal distribution of CpG sites according to FDR. TCGA (PRAD)-450K methylation array data was retrieved from the UCSC Xena browser^81^ to cross-check the DNA methylation patterns of the CpG sites.

### Characterization of differentially expressed lncRNAs in prostate cancer

A differential expression analysis of lncRNAs was conducted to identify the pivotal lncRNAs potentially involved in prostate cancer (PC). Concerning this, the lncRNA microarray dataset for PC was retrieved from the GEO database with the keyword “prostate cancer” while considering the following criteria for selecting the suitable datasets (i) the lncRNA dataset was restricted to “*Homo sapiens*”, (ii) the experiment type was specified to “non-coding RNA profiling by array”, and (iii) dataset having tumor and control samples. The dataset satisfying the aforementioned criteria was utilized for analyzing through the R package “limma”^82^ to identify the differentially expressed lncRNA (DElncRNA) between PC samples and control samples. FDR was computed through the Benjamini and Hochberg approach for adjusting the raw P-value to correct the occurrence of false positive results. lncRNAs showing the values of FDR < 0.05 and absolute log2FC (fold change) > 1.5 were defined as DElncRNAs. Based on the “HM450.hg38.manifest” and GENCODE v36 reference file, the genomic annotation of each lncRNA was done to figure out the DElncRNAs which contain DMCs within their promoter region and selected for further analysis. “ggplot2” R package was employed to generate a volcano plot to represent the expression pattern of lncRNAs. Moreover, “gplots” R package was used to visualize the expression profile of DElncRNA. Both the “ggplot2” and “gplots” R packages were accessed through the Galaxy server^83^.

### Correlation analysis between DNA methylation and lncRNA expression

A correlation analysis between the lncRNA expression and the methylation of DMCs was conducted to evaluate the impact of methylation on the expression of corresponding lncRNAs and to determine the relevant significant lncRNAs involved in PC. The expression value (quantified as FPKM values) of the selected DElncRNAs from 496 PC patients were extracted from the TCGA (PRAD)-RNAseq data by utilizing the UCSC Xena Browser. UCSC Xena is a scalable solution for the visualization and analysis of cancer genomics from large public data repositories like TCGA and the GDC as well as private datasets^81^. Similarly, the methylation value (quantified as beta values) of the selected DMCs from the same 496 PC patients were obtained from the TCGA (PRAD)-450K methylation array data through accessing the UCSC Xena Browser. The "cor.test" function of the R programming language^84^ was used to assess the Spearman's correlation coefficient (R) between the expression of the selected lncRNAs and the methylation level of the corresponding DMCs where significant threshold was fixed at an absolute value of R > 0.3 and P-value < 0.05. Only negatively correlated DElncRNA-DMC pairs were considered for further analysis as methylation in promoters is negatively correlate with corresponding gene expression^56^.

### Impact of aberrant DNA methylation and expression of DElncRNA on the clinical prognosis of prostate cancer patients

We examined the effect of aberrant methylation and expression of the identified respective CpG sites and DElncRNAs on the clinical prognosis of PC patients. Regarding this, clinical data (TCGA-PRAD) of 500 PC patients was downloaded from the GDC Data Portal^85^. GDC (Genomic Data Commons) is based on NCI (National Cancer Institute) generated data including genomic, proteomic, epigenomic, clinical and other uniformly processed data from The Cancer Genome Atlas (TCGA) and Therapeutically Applicable Research to Generate Effective Therapies (TARGET) programs to explore cancer research^85^. Initially, for each patient, we calculated the median of the expression and methylation value obtained in the previous step for the identified respective DElncRNAs and DMCs. Based on the median value the patients were then divided into low and high expression/methylation groups. Kaplan–Meier overall survival curve was generated to compare the clinical prognosis between the high and low expression subjects. Moreover, a univariate Cox regression analysis was carried out to investigate the association of the selected DElncRNAs and DMCs in the clinical prognosis of PC patients. Additionally, multivariate Cox regression analysis was also done to determine the association of the expression of these DElncRNAs with other clinical features of the patients (patients’ age and primary Gleason grade). The significance level was set at 95% CI (confidence interval) of HR (hazard ratio) ⊉ 1 and P-value < 0.05. The “survival”^86^ and “survminer”^87^ R packages was implemented for these analyses.

### Functional annotation and enrichment analysis

Gene Ontology-Molecular Function (GO-MF) and Gene Set Enrichment Analysis (GSEA) were performed to point out the molecular functions and the underlying biological processes in which the selected key DElncRNAs are involved. First, the protein-coding genes modulated by these DElncRNAs were identified through characterizing the lncRNA-mRNA interaction network by using the ENCORI pan-cancer analysis platform^88^. The lncRNA-mRNA interaction network was constructed by the Cytoscape software^89^. Afterwards, the identified protein-coding genes were evaluated to figure out their molecular functions by Enrichr web server^90^. The R package “circlize”^91^ was employed to generate a chord diagram for visualizing the gene-function link. The gene set enrichment analysis was carried out by the GSEA software^92^ to identify the biological processes associated with these DElncRNAs. For this, the log2FC value computed by the “limma” R package was used as the ranking metric for GSEA. In this analysis the gene sets were obtained from the canonical pathways sub-collection of the C2 collection in the Molecular Signatures Database (MSigDB)^93^.

## Supplementary Information

Supplementary Table S1.

Supplementary Table S2.

Supplementary Table S3.

## Data Availability

The datasets generated during and/or analyzed during the current study are available from the corresponding author on reasonable request.
